# Insecticidal Activity of *Photorhabdus luminescens* against *Drosophila suzukii*

**DOI:** 10.3390/insects9040148

**Published:** 2018-10-23

**Authors:** Rady Shawer, Irene Donati, Antonio Cellini, Francesco Spinelli, Nicola Mori

**Affiliations:** 1 Department of Plant Protection, Faculty of Agriculture (Saba Basha), Alexandria University, Alexandria 21531, Egypt; rady.shawer@alexu.edu.eg; 2 Department of Agronomy, Food, Natural Resources, Animals and the Environment (DAFNAE), University of Padova, Viale dell’Università 16, 35020 Legnaro (Pd), Italy; nicola.mori@unipd.it; 3 Department of Agricultural and Food Sciences (DISTAL), Alma Mater Studiorum—Università di Bologna, Viale Fanin 44, 40127 Bologna, Italy; i.donati@unibo.it (I.D.); antonio.cellini2@unibo.it (A.C.)

**Keywords:** entomopathogenic bacteria, biological control, invasive pests, Spotted Wing Drosophila, Bioassay

## Abstract

*Drosophila suzukii* causes considerable economic damage to small and thin-skinned fruits including cherry, blueberry, raspberry, grape and strawberry. Since it attacks fruits at the ripening stage, the use of chemical pesticides is limited due to the high risk of residues on fruit. Biological control is thus expected to play an essential role in managing this pest. The Gram-negative bacterium, *Photorhabdus luminescens* and its symbiotic *Heterorhabditis* spp. nematode have been shown to be highly pathogenic to insects, with a potential for replacing pesticides to suppress several pests. Insecticidal activity of *P. luminescens* at different bacterial cell concentrations and its cell-free supernatant were assessed against third-instar larvae and pupae of *D. suzukii* under laboratory conditions. *P. luminescens* suspensions had a significant oral and contact toxicity on *D. suzukii* larvae and pupae, with mortalities up to of 70–100% 10 days after treatment. Cell-free supernatant in the diet also doubled mortality rates of feeding larvae. Our results suggest that *P. luminescens* may be a promising candidate for biological control of *D. suzukii*, and its use in integrated pest management (IPM) programs is discussed.

## 1. Introduction

*Drosophila suzukii* (Matsumura) (Diptera Drosophilidae) is an invasive species that threatens soft fruit industries in America and Europe [1,2] through feeding on unripe and undamaged cherry, blueberry, raspberry, grape and strawberry [2,3,4,5,6], causing extensive economic losses [7]. Chemical pesticides are the main *D. suzukii* control methods [8], but their use has to be limited due to the high risk of residues on the fruit, insect resistance development and their negative impact on beneficial insects [9,10,11]. Alternative and more sustainable control strategies are therefore constantly being sought [12]. Biological control agents are expected to play an essential role, being a cost-effective and environmentally safe approach for the management of this pest [8]. Control approaches based on biological agents are thus highly recommended to establish effective and sustainable integrated pest management (IPM) programs.

Several commercially available biological agents including parasitoids [13,14], predators [15,16], nematodes [16,17], entomopathogenic fungi [16,18,19] and bacteria [20] have been evaluated against *D. suzukii* under laboratory conditions. Currently, formulations based on the entomopathogenic fungus *Beauveria bassiana* are allowed for treatment against *D. suzukii* on cherry, grapevine and strawberry in Europe [21]. Entomopathogenic bacteria, such as *Bacillus thuringiensis*, *Serratia* spp., *Pseudomonas entomophila*, *Burkholderia* spp., *Chromobacterium subtsugae*, *Xenorhabdus* and *Photorhabdus* spp. have also been studied for the practical control of different Diptera species [22,23,24,25].

*Photorhabdus luminescens*, a member of the Gammaproteobacteria, is a Gram-negative and mutualistic bacterium that lives in the gut of entomopathogenic nematodes belonging to the Heterorhabditidae family [26,27]. Both *P. luminescens* alone and its symbiotic *Heterorhabditis* spp. nematode are known to be highly pathogenic to insects [28]. Once the nematode infects an insect, *P. luminescens* is rapidly released into the haemocoel, where it secretes enzymes and high-molecular-weight toxin complexes (Tc) that disintegrate and bioconvert the body of the infected insect into nutrients, which can be consumed by both the nematode and bacterium [26].

A number of publications showed the efficacy of *P. luminescens* against larvae and pupae of tobacco hornworm, *Manduca sexta* (L.) (Lepidoptera: Sphingidae) [26], cotton leafworm *Spodoptera litura* (Fabricius) (Lepidoptera: Noctuidae) [29], diamondback moth *Plutella xylostella* (L.) (Lepidoptera: Plutellidae) [30], Colorado potato beetle *Leptinotarsa decemlineata* (Say) (Coleoptera: Chrysomelidae), tobacco whitefly *Bemisia tabaci* (Gennadius) (Hemiptera: Aleyrodidae) [31] and honeycomb moth *Galleria mellonella* (L.) (Lepidoptera: Pyralidae) larvae [12,29]. In addition, the discovery of Tc produced by *P. luminescens* has led to great interest in their development as replacements for *Bacillus thuringiensis* [32]. Tca, a high molecular weight insecticidal protein, was found to be orally toxic to both the Colorado potato beetle, *L. decemlineata*, and sweet potato whitefly, *B. tabaci* [31]. However, *P. luminescens* has never been tested against *D. suzukii*.

This study aimed to assess the insecticidal activity of *P. luminescens* against larvae and pupae of *D. suzukii* under laboratory conditions. Different concentrations of *P. luminescens* cell suspension were tested on pre-immaginal stages both for oral and contact toxicity. The cell free supernatant toxicity of the bacterium was also evaluated against larvae and pupae of *D. suzukii*. The effect of *P. luminescens* suspensions and cell-free supernatant was also tested on *D. suzukii* adults. Finally, the persistence of *P. luminescens* on cherry fruit was evaluated.

## 2. Materials and Methods

### 2.1. Insect Colony

Insects used in the experiments originated from adult *D. suzukii* collected from field infested cherry and grape fruits in Verona Province, North-Eastern Italy and maintained in the laboratory of the Department of Agronomy, Food, Natural Resources, Animals and the Environment, Padova University under controlled conditions. Male and female adults (mixed ages) were placed in plastic vials (Falcon type with 50 mL capacity, diameter 30 mm, length 115 mm) with a specific medium for *D. suzukii* rearing [33,34].

Diet components (75 g raw cornmeal, 17 g dry-yeast, 15 g sucrose, 12 g soybean meal, 5.6 g agar and water adjusted to 1000 mL) were thoroughly mixed and cooked for 20 min at about 100 °C, 5 mL propionic acid were then added at a temperature of less than 50 °C just before pouring 15 mL of medium into the plastic vials. Cultures were maintained in climate chambers held at 23 ± 1 °C, 70 ± 10% relative humidity and 16L:8D photoperiod. Wild *D. suzukii* were introduced into the colony on multiple occasions to ensure that the genetic make-up of the individuals screened in the laboratory was representative of the field population.

### 2.2. Bacteria Isolation and Culture Conditions

*P. luminescens* was isolated from the soil of the experimental farm of Bologna University (Cadriano, Lat: 44.548985, Long: 11.386292). For the isolation, *G. mellonella* larvae, purchased at a local fishing gear retailer, were placed in 15 mL plastic tubes closed with 1 mm-mesh nets [35]. Twenty tubes were buried at 10 cm depth in a set-aside plot of an orchard where chemical pesticide had not been directly sprayed in the last 12 months, to attract entomopathogenic nematodes naturally carrying *P. luminescens*. The tubes were left underground for 3 days. Successively, to isolate the bacterium, each *G. mellonella* larva was homogenized in 2 mL of sterile MgSO_4_ (10 mM). The homogenate was serially 10-fold diluted in the same buffer and 200 µL of each dilution was plated on a modified nutrient agar medium supplemented with 0.004% (*w*/*v*) triphenyltetrazolium chloride and 0.0025% (*w*/*v*) bromothymol blue (NBTA) [36] amended with cychloheximide (0.01% *w*/*v*) to prevent fungal contamination. The plates were incubated at 30 °C for 48 h. Colonies that absorbed bromothymol blue were selected and singularized onto MacConkey agar (Biolab) and tryptic soy broth (TSB) agar medium. The different isolates were identified as described by Liu et al. [37]. *P. luminescens* subsp. *akhurstii* (strain W14, DSM15138) was used as positive control from molecular identification. The strain was purchased from the Leibniz Institute DSMZ-German Collection of Microorganisms and Cell Cultures. Each identified isolate was stored at −80 °C in a cryotube containing TSB amended with 40% sterile glycerol.

The *P. luminescens* isolates were tested against *D. suzukii*. The bacterial suspension for the bioassay experiments on *D. suzukii* was prepared from *P. luminescens* culture grown in 200 mL of TSB incubated at 28 °C under moderate shaking (150 rpm) for 3 days. Bacterial concentration was estimated by optical density at 600 nm and adjusted to 3.5 × 10^8^ cells mL^−1^, which was higher than the average concentration (4 × 10^7^ cells mL^−1^) shown to be effective against larvae of *Aedes aegypti* [38] and *G. mellonella* [12]. The final concentration was also confirmed by 10-fold serial dilutions and plating on TSB agar medium. To obtain a bacterial supernatant containing toxic metabolites, the bacterial suspension at 3.5 × 10^8^ cells mL^−1^ concentration was centrifuged at 10,000× *g* for 10 min at room temperature. The obtained supernatant was filtrated with a 0.2 µm Millipore filter. The resulting filtrate supernatant was checked for sterility by placing a 100 µL aliquot on Luria–Bertani (LB) agar plates [34]. The supernatant was diluted 10-fold with TSB. The undiluted supernatant and 10-fold dilution were tested against larvae and pupae of *D. suzukii*.

### 2.3. Larval Oral Bioassay

For the oral bioassay [26,27] six bacterial concentrations (3.5 × 10^8^, 3.5 × 10^7^, 3.5 × 10^6^, 3.5 × 10^5^, 3.5 × 10^4^, 3.5 × 10^3^ cells mL^−1^) of *P. luminescens* and bacterial supernatants were evaluated against third-instar larvae of *D. suzukii*. Sterile *D. suzukii* diet (10 mL), amended with 1 mL of the *P. luminescens* suspensions or its supernatant was placed in 9 cm diameter Petri dishes and allowed to dry. Thus, each treatment corresponded to a 1:10 dilution of each bacterial suspension. Ten third-instar larvae were put in each Petri dish. All dishes were sealed with Parafilm before incubation at 25 °C for 4 days. TSB treatments acted as control. Each treatment had three replicates, and the experiments were independently repeated three times. Larvae mortality was recorded at 2 and 4 days after application (DAA). Larvae were considered dead if they did not move when lightly touched with a camelhair needle [38,39]. The development of surviving larvae into pupae and adults was then followed until 10 DAA given that the developmental periods from egg to adult is 9–10 days at a constant temperature of 25 °C [40,41]. Pupae were considered dead if they did not produce live adults. Percentage mortality was calculated for immature stages [larvae (4 DAA) and pupae (10 DAA)], and all life stages [larvae (4 DAA), pupae (10 DAA) and adults (10 DAA)] of *D. suzukii*.

### 2.4. Larval and Pupal Dipping Bioassay

Dipping bioassays were used for evaluating the insecticidal activity of *P. luminescens* at concentrations of 3.5 × 10^8^, 3.5 × 10^7^ and 3.5 × 10^6^ cells mL^−1^ against larvae and pupae of *D. suzukii*. The same procedures considered in the larvae-oral bioassay were followed in the dipping bioassays, except that larvae and pupae were fully immersed in bacterial cells for 30 s before being placed in Petri dishes, instead of adding *P. luminescens* to the diet. Controls were dipped in TSB. Survival was determined as development to subsequent stages. Larvae mortality was recorded 2 and 4 DAA. Mortality of emerged pupae and adults was assessed at 9 and 10 DAA, respectively. For pupal-dipping bioassay, mortality of both pupae and emerged adults at 9 DAA was recorded.

### 2.5. Pupal Direct-Spray Bioassay

Six bacterial-cell concentrations (3.5 × 10^8^, 3.5 × 10^7^, 3.5 × 10^6^, 3.5 × 10^5^, 3.5 × 10^4^, and 3.5 × 10^3^ cells mL^−1^) of *P. luminescens* and bacterial supernatants were tested against mature pupae of *D. suzukii* (7 days old). Ten pupae were placed in Petri dishes and the solution was applied topically (100 µL) under a stereomicroscope [42]. Thus, each pupa was treated with 3.5 × 10^2^ to 3.5 × 10^7^ bacterial cells. Controls were treated by TSB. Each treatment was replicated 3 times. Petri dishes were then incubated as previously described. Mortality of pupae and emerged adults at 9 DAA was recorded.

### 2.6. Adult Insect Bioassay

*P. luminescens* was allowed to grow in the LB broth, then it was precipitated by centrifugation (5000× *g*, 10 min) and resuspended in sterile 10 mM MgSO_4_ solution to a final concentration of 2 × 10^6^, 2 × 10^4^, and 2 × 10^2^ cells mL^−1^. The supernatant was filter-sterilised with a 0.2 µm Millipore filter.

The bacterial suspensions and the culture supernatant were tested against adults of *D. suzukii* (2–3 weeks old). After mixing 1:1 (*v*/*v*) with a 20 mL L^−1^ agar solution maintained at 50 °C, the bacterial suspension or supernatant were spread on the walls of a 50 mL Falcon tube. Five to ten insect flies were introduced into the tube, together with about 1 g of freshly-cut cherry slices, and closed with cotton tissue to allow for air exchange. Controls were treated with sterile MgSO_4_ solution (to evaluate the effect of live bacteria suspensions) and LB medium (to evaluate the effect of solutes dissolved in the supernatant). Each treatment was replicated 3 times. The tubes were incubated as previously described, and mortality was recorded every two days until 9 DAA.

### 2.7. Bacterial Persistence on Cherry Fruit

Cherry fruit were surface-sterilized by washing in 1.5% NaOCl for 10 min. Subsequently, the fruit was rinsed twice in sterile water, and inoculated by 1-min dipping in a bacterial suspension (1.1 × 10^8^ cells mL^−1^) in 10 mM MgSO_4_. After inoculation, the fruit was allowed to dry under laminar air flow, and then maintained at room temperature in a sterile jar allowing gas exchange.

Three fruit samples were taken 0, 3, 24, 48 and 96 h post inoculation. Each cherry was singularly washed in 10 mL MgSO_4_ solution (30 min, 120 rpm shaking), and serial tenfold dilutions of the wash were plated on LB-Agar plates amended with 100 mg L^−1^ cycloheximide, to determine the bacterial concentration. Before inoculation, a fruit sample was subjected to the same process to verify the sample sterilization.

### 2.8. Data Analysis

Bioassay data were analyzed by one-way analysis of variance (ANOVA) followed by means separation with Fisher’s least significant difference (LSD) test, using the appropriate models for a completely randomized design (laboratory bioassays) (SAS Institute, Cary, NC, USA, 2010). Percentages of adult mortality were transformed to arcsine [sqrt (%mortality)] before analysis to stabilize variance and reported means were back-transformed to percentages for presentation. Data were expressed as the mean and confidence intervals (CI) of all the replicates from the three experimental repetitions (*n* = 9). Differences were considered significant at *p* < 0.05 level. Abbott’s formula [43] was used to correct for control mortality (efficacy).

## 3. Results

### 3.1. Larval Oral Bioassay

**Figure 1:**
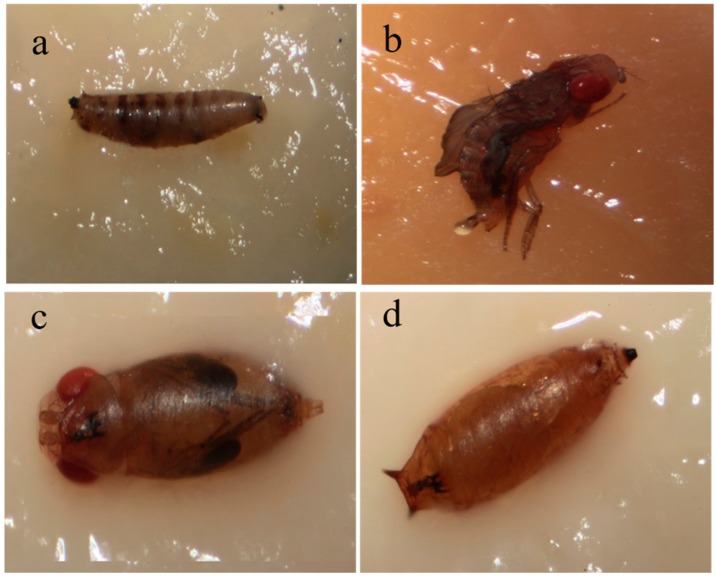
Appearance of *Drosophila suzukii* specimens after treatment with *Photorhabdus luminescens*. (**a**) Dead larva that fed on diet containing *P. luminescens*. (**b**) Dead adult, fed as a larva on diet containing *P. luminescens*. (**c**) Deformed adult, dipped in *P. luminescens* solution as a pupa. (**d**) Live *D. suzukii* female pupa, dipped as a larva in tryptic soy broth (TSB) medium (control).

The influence of a diet containing *P. luminescens* cells on *D. suzukii* larval mortality and subsequent life stages (pupae and adults) was investigated (Figure 1a,b, Table 1). There were no significant (*F* = 0.87; df = 6; *p* = 0.5425) differences between all treatments on larval mortality 2 DAA; however, the three highest concentrations (3.5 × 10^8^, 3.5 × 10^7^, 3.5 × 10^5^ cells mL^−1^) caused higher mortality than the control. The number of dead larvae was significantly (*F* = 3.54; df = 6; *p* = 0.0240) affected by treatments at 4 DAA: the best efficacy (36.7%) was shown by the 3.5 × 10^8^ cells mL^−1^ concentration, followed by 3.5 × 10^7^ cells mL^−1^ (33.3%). The number of emerged pupae at 10 DAA was not significantly suppressed by their larvae feeding on *P. luminescens* at tested concentrations (*F* = 0.62; df = 6; *p* = 0.7100). All treatments significantly reduced emerged adult numbers at 10 DAA compared to the control, with the highest reduction (50.0%) in 3.5 × 10^8^ cells mL^−1^ treatment (*F* = 2.13; df = 6; *p* = 0.0114). *D. suzukii* immature individuals were significantly (*F* = 4.08; df = 6; *p* = 0.0141) affected by treatments. The 3.5 × 10^8^ and 3.5 × 10^7^ cells mL^−1^ concentrations provided significant mortality in immature stages of 46.7% and 36.7%, respectively. Overall, treatments were highly significant (*F* = 12.53; df = 6; *p* < 0.0001), causing greater mortality of all *D. suzukii* life stages (larvae at 4 DAA, pupae and adults at 10 DAA) than the control, with excellent activity (above 90%) in 3.5 × 10^8^ and 3.5 × 10^7^ cells mL^−1^ treatments, while the others showed fairly good efficacy of more than 60%.

The insecticidal activity of *P. luminescens* supernatants was also tested against *D. suzukii* larvae and their subsequent life stages (pupae and adults) (Table 2). Supernatant treatments significantly reduced the number of larvae at 4 DAA (*F* = 5.55; df = 2; *p* = 0.0197), immature stages at 10 DAA (*F* = 10.50; df = 2; *p* = 0.0023), and all individuals at 10 DAA (*F* = 14.98; df = 2; *p* = 0.0005) compared to the control. The bacterial supernatant obtained from *P. luminescens* concentration 3.5 × 10^8^ cells mL^−1^ was significantly effective, providing 24%, 44%, and 74% mortality of larvae at 4 DAA, immature stages at 10 DAA, and all individuals at 10 DAA, respectively. When the concentration of bacterial supernatant was 10-fold diluted, the insecticidal performance did not significantly decrease.

### 3.2. Larval and Pupal Dipping Bioassay

In these bioassays, the potential activity of *P. luminescens* was tested on *D. suzukii* larvae and pupae (Figure 1c,d), while the supernatants were not evaluated. For larval-dipping bioassay (Table 3), none of the tested *P. luminescens* concentrations were able to significantly decrease the number of treated larvae at 2 (*F* = 0.25; df = 3; *p* = 0.8592) and 4 (*F* = 1.07; df = 3; *p* = 0.4158) DAA. However, higher mortality than the control was observed in the 3.5 × 10^7^ and 3.5 × 10^8^ cells mL^−1^ treatments at 4 DAA. A significant decrease in the number of emerged pupae that were previously treated as larvae was caused in the treatment 3.5 × 10^8^ cells mL^−1^ at 9 DAA compared to the control (*F* = 3.09; df = 3; *p* = 0.0481), showing pupal efficacy of 54%. No significant differences appeared in the mortality of emerged adults at 10 DAA (*F* = 1.10; df = 3; *p* = 0.4033). By the tenth day, *P. luminescens* significantly (*F* = 4.25; df = 3; *p* = 0.0451) reduced the number of treated immature stages, with the highest activity (64%) observed at 3.5 × 10^8^ cells mL^−1^ concentration. *D. suzukii* individuals at all life stages were significantly (*F* = 5.40; df = 3; *p* = 0.0252) affected by the tested concentrations. High efficacy (82%) was provided with a concentration of 3.5 × 10^8^ cells mL^−1^; while fairly good efficacy (50%) was shown with 3.5 × 10^7^.

For the pupal dipping bioassay (Table 4), no significant differences were observed in the number of pupae (*F* = 2.83; df = 3; *p* = 0.1062) or emerged adults (*F* = 0.64; df = 3; *p* = 0.6116) at 9 DAA, however, all *P. luminescens* treatments caused higher percentage mortality than the control. Generally, all treatments caused greater mortality of pupa and adult *D. suzukii* individuals than the control due to their pupae being dipped in different bacterial concentrations, with significant mortality (≥70%) at 3.5 × 10^8^ and 3.5 × 10^7^ cells mL^−1^.

### 3.3. Pupal Direct-Spray Bioassay

In the pupal direct-spray bioassay (Table 5), significant reductions in live pupae numbers at 9 DAA were caused by all concentrations compared to the control (*F* = 4.38; df = 6; *p* = 0.0107), while the number of emerged adults was not significantly affected (*F* = 2.01; df = 6; *p* = 0.1326) even if the treatments caused a higher percentage mortality than the control. Overall, significant differences (*F* = 32.96; df = 6; *p* < 0.0001) in the mortality of pupating and adult *D. suzukii* individuals were caused in all treatments as a result of pupae being sprayed with different bacterial concentrations, with high efficacy (100%) at 3.5 × 10^8^ and 3.5 × 10^7^ cells mL^−1^.

Regarding supernatant applications, the treated pupae were not significantly affected by either undiluted supernatant or the 10-fold dilution (*F* = 1.50; df = 2; *p* = 0.2621), even if mortality was higher than the control (Table 2). A significant effect of *P. luminescens* supernatant (*F* = 10.29; df = 2; *p* = 0.0025) appeared against the emerged *D. suzukii* adults at 9 DAA. Overall, a 74% cumulative mortality in all individuals was significant (*F* = 22.56; df = 2; *p* < 0.0001) in the undiluted supernatant treatment; the supernatant 10-fold dilution treatment was less effective (Table 2).

### 3.4. Adult Insect Bioassay

**Figure 2:**
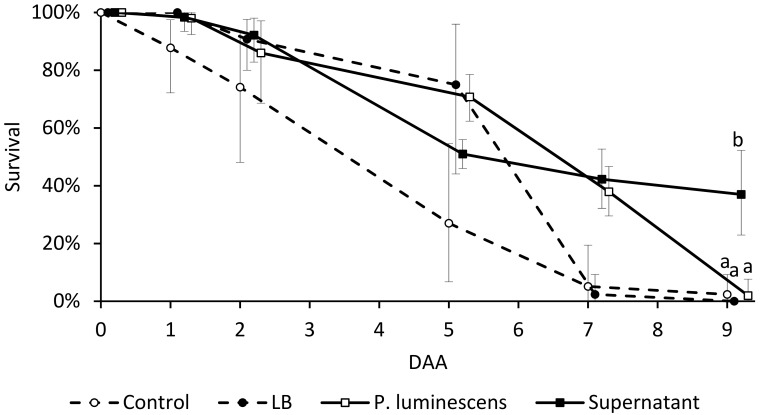
*Drosophila suzukii* adult survival (average and CI from *n* = 3 samples) in tubes treated with *Photorhabdus luminescens* (10^6^ cells mL^−1^) or *P. luminescens* culture supernatant over 9 days after application (DAA). Survival rates were compared to 10 mM MgSO_4_ (control) or axenic Luria–Bertani (LB) medium. Different letters show significant differences among different treatments for each day, according to LSD test; *p* < 0.05.

The inclusion of *D. suzukii* adult insects in tubes colonized by *P. luminescens* did not reduce their survival rate. Spreading the bacterial supernatant in the tube significantly increased insect survival 9 DAA (*F* = 3.19; df = 12; *p* = 0.04618) in comparison to any other treatment (Figure 2): at that time point, mortality in the supernatant-treated samples was 65%, compared to approximately 95% in the controls.

### 3.5. Bacterial Persistence on Cherry Skin

**Figure 3:**
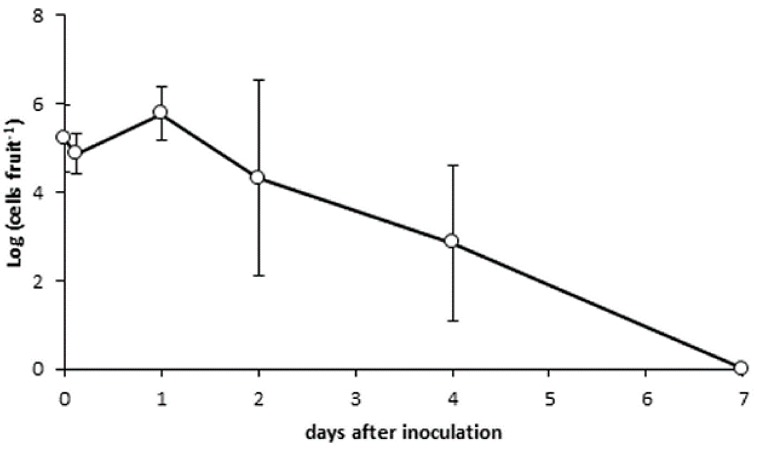
Survival of *Photorhabdus luminescens* on cherry fruit over 7 days after inoculation.

*P. luminescens* maintained similar population levels (approx. 10^5^ bacterial cells per fruit) over four days after inoculation. The bacterium was undetectable one week after inoculation (Figure 3).

## 4. Discussion

All of the currently known *Photorhabdus* spp. display entomopathogenic properties, which are expressed upon colonization of the target insect by nematode species forming a symbiosis with the bacterium. Isolates from human clinical cases, initially recognized as *P. luminescens*, were subsequently reassigned to the species *P. asymbiotica* basing on molecular taxonomy and pathogenetic potential [44]. Thus, *P. luminescens* is safe for agricultural operators and consumers. This study provides new information for the possible use of *P. luminescens* to control *D. suzukii* larvae and pupae. Indeed, the bacterium caused a high mortality of pre-immaginal stages through both oral and contact toxicity. Interestingly, a significant efficacy was found also with very low bacteria concentration. In the oral bioassay, the bacterium increased the mortality of larvae and reduced the viability of the emerging adults even at a concentration of 3.5 × 10^2^ cells mL^−1^.

The larval bioassays showed significant insecticidal activities of *P. luminescens* against all *D. suzukii* development stages with mortality ranging between 86.7% and 100%. Parallel findings were obtained by Rahoo et al. [12] on *Galleria mellonella* larvae using *P. luminescens* at a concentration of 4 × 10^7^ cells mL^−1^ and by da Silva et al. [38] on *Aedes aegypti* L. (Diptera: Culicidae) larvae. Within 24 h of being given the diet the treated larvae of *D. suzukii* stopped feeding and produced less faeces than usual. After cessation of feeding, larvae body colour changed from green to yellow or black (Figure 1a). However, larvae can survive for several days without feeding. This explains why the significant effect of *P. luminescens* bacteria began to appear 4 DAA. The bacteria seem to be more effective if they are swallowed; oral toxicity was about 37% vs. 10% contact toxicity. In our study, the bacterial supernatant caused 24% larval mortality, and 74% cumulative mortality of all *D. suzukii* development stages, confirming that toxins secreted by these bacteria have the potential to kill insects. The results agree with those previously obtained by Blackburn et al. [26] on larvae of *Manduca sexta* after they were fed a diet treated with 1 µg of Tca, a toxin complex secreted by *P. luminescens*. Moreover, the injection of 100 cells of *P. luminescens* (K^−1^) isolate was found to be lethal for *G. mellonella* larvae in 48 h. Likewise, the bacterial toxin secreted into the growth medium as the culture supernatant killed the insect in 48 h [29].

For the pupal bioassay, all concentrations were able to reduce pupae individuals compared to the control, achieving mortality ranges from 43.3–63.3%. A total mortality of 100% and 73.33% was caused by 3.5 × 10^8^ cells mL^−1^ following pupal direct-spray and dipping bioassays, respectively. According to our visual observations, the treatment induced a high frequency of deformed adults (Figure 1c) emerging from pupae treated with *P. luminescens* cells (data not shown). Most of those deformed adults were unable to fully emerge and pursue their normal life stages. No deformed adults were noted in the control. Treatments (either with bacterial cells, or with toxin-containing supernatant) applied to adult insects had no insecticidal effect. This work did not investigate whether the offspring of treated adults is affected by *P. luminescens*. Neither the axenic bacterial medium (LB), nor the bacterial supernatant showed any toxic effects on *D. suzukii* adults. Since the bacteria growing medium share several components with the media used to rear insects, it may actually represent a nutrient source for the insects.

So far, the commercial use of these bacterial endosymbionts is related to the employment of the nematode species with which they are associated [20]. These results show the possibility of the use of *P. luminescens* in new IPM strategies to control *D. suzukii*. Bacterial suspension of *P. luminescens* has been used as a topical pesticide against a wide range of insects like aphids and mealybugs [45,46]. Considering that *D. suzukii* eggs are laid in the ripe fruits, the application should be done preventively at the beginning of oviposition (against eggs near surface of fruit). To apply the *P. luminescens* against *D. suzukii* larvae (first instar), further investigation is required to evaluate the ability of bacterial suspensions to penetrate through fruit skin. Topical application of biological agents on fruit is advantageous in terms of safety, potency and specificity compared to chemicals. These aspects, together with bio-degradability, may suggest a large and competitive market for *P. luminescens*-based biocontrol products. The high efficacy against larvae may also help in reducing the environmental population of the pest. In fact, bacteria can be sprayed also on wild host fruit plants that may act as pest reservoirs. Bacteria application could also be an economic alternative to clean harvest since the treatments of waste or non-harvested fruit may prevent the emergence of new pest generations. These kinds of application on fruit are particularly promising since, according to our data, a single bacterial application may maintain a sufficiently high population on fruit for at least 5 days. Moreover, *P. luminescens* acts against larvae at very low concentrations ranging between 10^2^ and 10^3^ cells mL^−1^. Therefore, a natural spread of *P. luminescens*, up to an effective protective population, is likely to occur from treated plants to neighbouring ones.

Entomopathogenic bacteria can be used as stand-alone products for pest management in organic farming, their use in rotation or combination with chemicals is strongly encouraged to achieve full efficacy and eco-sustainability. Many studies have highlighted compatibility and synergistic effects of entomopathogenic bacteria and chemical substances [47,48]. For example, the conjugation of a pesticide that provides a rapid knock-down of adult population, followed by a bioinsecticide that targets larvae in fruit, may provide a successful strategy optimizing treatment efficacy and coverage. This work shows that *P. luminescens* is a promising tool for the containment of *D. suzukii* population. However, for its technological application in open field conditions, further studies are needed to assess the efficacy and formulation stability of products based on bacterial suspensions in different crops and environmental conditions.

In an alternative to topical applications of *P. luminescens*, other delivery methods should be investigated for the practical use in field conditions. For example, *P. luminescens* stabilized cells can be added to specific lures in order to obtain a horizontal spread of the pathogen from one individual to another. The use of stabilized, liophylized cells will also allow to recycle the purified cultural supernatant, which in our experiments showed a good degree of efficacy, for the application as a topical bioinsecticide.

To increase the efficacy of this bioinsecticide and make it comparable to chemical pesticides, it is essential to achieve a rapid control of the target pest. There is little information on conversion of bioagents or their by-products to enhance the toxicological properties. The efficacy of extracellular secretions of *P. luminiscens* could be improved by downsizing the particles through nanotechnology [49]. The results of Ramesh et al. [49] revealed that is possible to enhance the insecticidal property with a faster mortality of the target insect, attributed to higher penetration power of the nanoparticle, which acts as a small carrier of the toxin complex rapidly killing the insect.

## 5. Conclusions

Although agrochemicals are still the most convenient method for controlling pests, the use of alternative methods is increasing due to the negative side effects of pesticides. Even if a certain degree of prudence is recommended in the use of these biological pest control agents, entomopathogenic bacteria or their by-products could be a valid alternative or combined method to reduce the intensive use of xenobiotic chemicals, resulting in a significant environmental benefit.

## Figures and Tables

**Table 1:** Mortality of *Drosophila suzukii* larvae, pupae and adults following the *Photorhabdus luminescens* larval-oral bioassay.

Concentration (Cells mL^−1^)	Larvae		Pupae	Adults	Immatures	Total Individuals
	2 DAA	4 DAA	10 DAA	10 DAA	10 DAA	10 DAA
3.5 × 10^8^	10.0 (10.0–10.0) a	36.7 (8.0–65.0) a	10.0 (−33.0–53.0) a	50.0 (25.2–74.8) a	46.7 (8.7–84.6) a	96.7 (82.3–111) a
3.5 × 10^7^	3.3 (−11–17.6) a	33.3 (−4.6–71.3) ab	3.3 (−11.0–177.0) a	53.3 (−22.6–129.0) a	36.7 (−15–88.4) a	90.0 (65.1–114.8) ab
3.5 × 10^6^	6.7 (−22–35.4) a	10.0 (−14.8–34.8) c	10.0 (−14.8–34.8) a	53.3 (−22.6–129.0) a	20.0 (−14.8–34.8) ab	73.3 (44.6–102.0) bc
3.5 × 10^5^	6.7 (−22–35.4) a	13.3 (−1.0–27.7) bc	0.0 (0.0–0.0) a	53.3 (1.6–105.0) a	13.3 (−1.0–27.7) b	66.7 (9.3–124.0) c
3.5 × 10^4^	0.0 (0.0–0.0) a	6.7 (−22.0–35.4) c	6.7 (−22.0–35.4) a	46.7 (18.0–75.4) a	13.3 (−15.4–42.0) b	60.0 (60.0–60.0) c
3.5 × 10^3^	0.0 (0.0–0.0) a	10.0 (−33.0–53.0) c	3.3 (−11.0–17.7) a	46.7 (8.7–84.6) a	13.3 (−24.6–51.3) b	60.0 (10.3–109.7) c
Control	3.3 (−11.0–17.7) a	6.7 (−7.6–21.0) c	0.0 (0.0–0.0) a	6.7 (−7.7–21.0) b	6.7 (−7.7–21.0) b	13.3 (−1.0–27.7) d

DAA = days after application. Means and CI were obtained from *n* = 9 samples. Values followed by the same letter(s) within columns are not significantly different according to LSD test for *p* < 0.05.

**Table 2:** Mortality of *Drosophila suzukii* larvae, pupae and adults following treatment with *Photorhabdus luminescens* supernatant at 3rd instar larvae or pupae stages.

**Treatment on Larvae**	**Larvae,** **4 DAA**	**Immatures,** **10 DAA**	**Total Individuals,** **10 DAA**
Bacterial supernatant	24 (11.6–32.4) a	44 (28.4–55.6) a	74 (51.6–92.4) a
Supernatant 10-fold dilution	12 (−1.6–25.6) b	18 (1.8–34.2) b	34 (15.2–52.8) b
Control	4 (−2.8–10.8) b	12 (1.6–22.4) b	28 (17.6–38.4) b
**Treatment on Pupae**	**Pupae,** **9 DAA**	**Adults,** **9 DAA**	**Total Individuals,** **9 DAA**
Bacterial supernatant	38 (17.6–58.4) a	36 (15.2–56.8) a	74 (59.8–88.2) a
Supernatant 10-fold dilution	26 (9.3–42.7) a	16 (9.2–22.8) b	42 (25.8–58.2) b
Control	26 (19.2–32.8) a	6 (−0.8–12.8) b	32 (26.4–37.6) b

DAA = days after application. For each development stage, means and CI were obtained from *n* = 9 samples. Values followed by the same letter(s) are not significantly different according to LSD test for *p* < 0.05.

**Table 3:** Mortality of *Drosophila suzukii* larvae, pupae and adults following dipping the larvae in *Photorhabdus luminescens* at different concentrations.

Concentration (Cells mL^−1^)	Larvae		Pupae	Adults	Immatures	Total Individuals
	2 DAA	4 DAA	9 DAA	10 DAA	10 DAA	10 DAA
3.5 × 10^8^	6.7 (−7.7–21.0) a	10.0 (10.0–10.0) a	60.0 (−5.0–125.7) a	16.7 (−35.0–68.4) a	70.0 (4.3–135.7) a	86.7 (58.0–115.4) a
3.5 × 10^7^	3.3 (−11.0–17.7) a	10.0 (−14.0–34.8) a	36.7 (22.3–51) ab	16.7 (2.3–31.0) a	46.7 (8.7–84.6) ab	63.3 (11.6–115) ab
3.5 × 10^6^	3.3 (−11.0–17.7) a	3.3 (−11.0–17.7) a	16.7 (−55.0–88.4) b	30.0 (−13.0–73.0) a	20.0 (−45.7–85.7) b	50.0 (−15.7–115.7) bc
Control	3.3 (−11.0–17.7) a	3.3 (−11.0–17.7) a	13.3 (−1.0–27.7) b	10.0 (10.0–10.0) a	16.7 (2.3–31) b	26.7 (12.3–41.0) c

DAA = days after application. Means and CI were obtained from *n* = 9 samples. Values followed by the same letter(s) within columns are not significantly different according to LSD test for *p* < 0.05.

**Table 4:** Mortality of *Drosophila suzukii* pupae and emerged adults 9 days after dipping the pupae in *Photorhabdus luminescens* at different concentrations.

Concentration (Cells mL^−1^)	Pupae	Adults	Total Individuals
3.5 × 10^8^	46.7 (32.3–61.0) a	26.7 (−45.0–98.4) a	73.3 (16.0–130.7) a
3.5 × 10^7^	46.7 (8.7–84.6) a	23.3 (−39.2–85.8) a	70.0 (4.3–135.7) a
3.5 × 10^6^	43.3 (5.4–81.3) ab	23.3 (9.0–37.7) a	66.7 (15.0–118.4) ab
Control	23.3 (9.0–37.7) b	6.7 (−7.7–21.0) a	30.0 (5.2–54.8) b

Means and CI were obtained from *n* = 9 samples. Values followed by the same letter(s) within columns are not significantly different according to LSD test for *p* < 0.05.

**Table 5:** Mortality of *Drosophila suzukii* pupae and adults, 9 days after spraying the pupae with *Photorhabdus luminescens* at different concentrations.

Concentration (Cells mL^−1^)	Pupae	Adults	Total Individuals
3.5 × 10^8^	63.3 (6.0–120.7) a	36.7 (−20.7–94.0) a	100.0 (100.0–100.0) a
3.5 × 10^7^	50.0 (−15.7–115.7) a	50.0 (−15.7–115.7) a	100.0 (100.0–100.0) a
3.5 × 10^6^	63.3 (34.6–92.0) a	30.0 (5.2–54.8) ab	93.3 (79.0–107.7) ab
3.5 × 10^5^	63.3 (49.0–77.7) a	26.7 (12.3–41.0) ab	90.0 (65.2–114.8) ab
3.5 × 10^4^	56.7 (42.3–71.0) a	30.0 (5.2–54.8) ab	86.7 (58.0–115.4) ab
3.5 × 10^3^	50.0 (7.0–93.0) a	33.3 (19.0–47.7) ab	83.3 (45.4–121.3) b
Control	10.0 (10.0–10.0) b	10.0 (10.0–10.0) b	20.0 (20.0–20.0) c

Means and CI were obtained from *n* = 9 samples. Values followed by the same letter(s) within columns are not significantly different according to LSD test for *p* < 0.05.

## References

[1] Cini A., Ioratti C., Anfora G. (2012). A review of the invasion of *Drosophila suzukii* in Europe and a draft research agenda for integrated pest management. Bull. Insectol..

[2] Walsh D.B., Bolda M.P., Goodhue R.E., Dreves A.J., Lee J., Bruck D.J., Walton V.D., O’Neal S.D., Zalom F.G. (2011). *Drosophila suzukii* (Diptera: Drosophilidae): Invasive pest of ripening soft fruit expanding its geographic range and damage potential. J. Integr. Pest Manag..

[3] Bellamy D.E., Sisterson M.S., Walse S.S. (2013). Quantifying host potentials: Indexing postharvest fresh fruits for spotted wing drosophila, *Drosophila suzukii*. PLoS ONE.

[4] Lee J.C., Bruck D.J., Curry H., Edwards D., Haviland D.R., Van Steenwyk R.A., Yorgey B.M. (2011). The susceptibility of small fruits and cherries to the spotted-wing drosophila, *Drosophila suzukii*. Pest Manag. Sci..

[5] Lee J.C., Bruck D.J., Dreves A.J., Ioriatti C., Vogt H., Baufeld P. (2011). In focus: Spotted wing drosophila, *Drosophila suzukii*, across perspectives. Pest Manag. Sci..

[6] Sasaki M., Sato R. (1995). Bionomics of the cherry drosophila, *Drosophila suzukii* Matsumura (Diptera: Drosophilidae) in Fukushima prefecture (Japan). Ann. Rep. Soc. Plant Prot. North Jpn..

[7] Goodhue R.E., Bolda M., Farnsworth D., Williams J.C., Zalom F.G. (2011). Spotted wing drosophila infestation of California strawberries and raspberries: Economic analysis of potential revenue losses and control costs. Pest Manag. Sci..

[8] Haye T., Girod P., Cuthbertson A.G.S., Wang X.G., Daane K.M., Hoelmer K.A., Baroffio C., Zhang J.P., Desneux N. (2016). Current SWD IPM tactics and their practical implementation in fruit crops across different regions around the world. J. Pest Sci..

[9] Desneux N., Decourtye A., Delpuech J.-M. (2007). The sublethal effects of pesticides on beneficial arthropods. Annu. Rev. Entomol..

[10] Haviland D.R., Beers E.H. (2012). Chemical control programs for *Drosophila suzukii* that comply with international limitations on pesticide residues for exported sweet cherries. J. Integr. Pest Manag..

[11] Stark J.D., Banks J.E. (2003). Population-level effects of pesticides and other toxicants on arthropods. Annu. Rev. Entomol..

[12] Rahoo A.M., Mukhtar T., Gowen S.R., Pembroke B. (2011). Virulence of entomopathogenic bacteria *Xenorhabdus bovienii* and *Photorhabdus luminescens* against *Galleria mellonella* larvae. Pak. J. Zool..

[13] Miller B., Anfora G., Buffington M., Daane K.M., Dalton D.T., Hoelmer K.M., Rossi Stacconi M.V., Grassi A., Ioriatti C., Loni A. (2015). Seasonal occurrence of resident parasitoids associated with *Drosophila suzukii* in two small fruit production regions of Italy and the USA. Bull. Insectol..

[14] Wang X.-G., Stewart T.J., Biondi A., Chavez B.A., Ingels C., Caprile J., Grant J.A., Walton V.M., Daane K.M. (2016). Population dynamics and ecology of *Drosophila suzukii*. J. Pest Sci..

[15] Renkema J.M., Telfer Z., Gariepy T., Hallett R.H. (2015). *Dalotia coriaria* as a predator of *Drosophila suzukii*: Functional responses, reduced fruit infestation and molecular diagnostics. Biol. Control.

[16] Woltz J., Donahue K., Bruck D., Lee J. (2015). Efficacy of commercially available predators, nematodes and fungal entomopathogens for augmentative control of *Drosophila suzukii*. J. Appl. Entomol..

[17] Cuthbertson A.G.S., Collins D.A., Blackburn L.F., Audsley N., Bell H.A. (2014). Preliminary screening of potential control products against *Drosophila suzukii*. Insects.

[18] Cuthbertson A.G.S., Audsley N. (2016). Further screening of entomopathogenic fungi and nematodes as control agents for *Drosophila suzukii*. Insects.

[19] Naranjo-Lázaro J.M., Mellín-Rosas M.A., González-Padilla V.D., Sánchez-González J.A., Moreno-Carrillo G., Arredondo-Bernal H.C. (2014). Susceptibility of *Drosophila suzukii* Matsumura (Diptera: Drosophilidae) to entomophatogenic fungi. Southwest. Entomol..

[20] Wise J.C., Van Woerkom A.H., Wheeler C.E., Isaacs R. (2018). IR-4 Attract N Kill tactics for control of spotted wing drosophila in blueberry. Arthropod Manag. Tests.

[21] EU-Pesticides-Database (2018). European Union Pesticides Database. http://ec.europa.eu/food/plant/pesticides/eu-311pesticides-database.

[22] Mahar A., Jan N., Mahar G.M., Mahar A.Q. (2008). Control of insects with entomopathogenic bacterium *Xenorhabdus nematophila* and its toxic secretions. Int. J. Agric. Biol..

[23] Ruiu L. (2015). Insect pathogenic bacteria in integrated pest management. Insects.

[24] Fanning P.D., Grieshop M.J., Isaacs R. (2018). Efficacy of biopesticides on spotted wing drosophila, *Drosophila suzukii* Matsumura in fall red raspberries. J. Appl. Entomol..

[25] Cahenzli F., Strack T., Daniel C. (2018). Screening of 25 different natural crop protection products against *Drosophila suzukii*. J. Appl. Entomol..

[26] Blackburn M., Golubeva E., Bowen D., Ffrench-Constant R.H. (1998). A novel insecticidal toxin from *Photorhabdus luminescens*, toxin complex a (Tca), and its histopathological effects on the midgut of *Manduca sexta*. Appl. Environ. Microbiol..

[27] Waterfield N., Dowling A., Sharma S., Daborn P.J., Potter U., Ffrench-Constant R.H. (2001). Oral toxicity of *Photorhabdus luminescens* W14 toxin complexes in *Escherichia coli*. Appl. Environ. Microbiol..

[28] Guo L., Fatig R.O., Orr G.L., Schafer G.W., Strickland J.A., Sukhapinda K., Woodsworth A.T., Petell J.K. (1999). *Photorhabdus luminescens* W-14 insecticidal activity consists of at least two similar but distinct proteins. Purification and characterization of Toxin A and Toxin B. J. Biol. Chem..

[29] Rajagopal R., Bhatnagar R.K. (2002). Insecticidal toxic proteins produced by *Photorhabdus luminescens akhurstii*, a symbiont of *Heterorhabditis indica*. J. Nematol..

[30] Mahar A.N., Jan N.D., Mahar A.Q., Mahar G.M., Hullio M.H., Lanjar A.G. (2008). Efficacy of entomopathogenic bacterium *Photorhabdus luminescens* and its metabolites against diamondback moth *Plutella xylostella* larvae on chinese cabbage and artificial diet. Pak. J. Nematol..

[31] Blackburn M.B., Domek J.M., Gelman D.B., Hu J.S. (2005). The broadly insecticidal *Photorhabdus luminescens* toxin complex a (Tca): Activity against the Colorado potato beetle, *Leptinotarsa decemlineata*, and sweet potato whitefly, *Bemisia tabaci*. J. Insect Sci..

[32] Dowling A., Waterfield N.R. (2007). Insecticidal toxins from *Photorhabdus bacteria* and their potential use in agriculture. Toxicon.

[33] Shawer R., Tonina L., Tirello P., Duso C., Mori N. (2018). Laboratory and field trials to identify effective chemical control strategies for integrated management of *Drosophila suzukii* in European cherry orchards. Crop Prot..

[34] Tonina L., Mori N., Giomi F., Battisti A. (2016). Development of *Drosophila suzukii*. J. Pest Sci..

[35] Kehres J., Denon D., Mauléon H. (2001). A simple technique to estimate, in situ, population densities of an entomopathogenic nematode (*Heterorhabditis indica*) in sandy soils. Nematology.

[36] Sicard M., Hering S., Schulte R., Gaudriault S., Schulenburg H. (2007). The effect of *Photorhabdus luminescens* (Enterobacteriaceae) on the survival, development, reproduction and behaviour of *Caenorhabditis elegans* (Nematoda: Rhabditidae). Environ. Microbiol..

[37] Liu J., Berry R.E., Blouin M.S. (2001). Identification of symbiotic bacteria (*Photorhabdus* and *Xenorhabdus*) from the entomopathogenic nematodes *Heterorhabditis marelatus* and *Steinernema oregonense* based on 16S rDNA sequence. J. Invertebr. Pathol..

[38] da Silva O.S., Prado G.R., da Silva J.L.R., Silva C.E., da Costa M., Heermann R. (2013). Oral toxicity of *Photorhabdus luminescens* and *Xenorhabdus nematophila* (Enterobacteriaceae) against *Aedes aegypti* (Diptera: Culicidae). Parasitol. Res..

[39] World Health Organization (1981). Instructions for Determining the Susceptibility or Resistance of Mosquito Larvae to Insecticides. http://apps.who.int/iris/bitstream/handle/10665/69615/WHO?sequence=1.

[40] Kanzawa T. (1939). Studies on *Drosophila suzukii* Mats. Kofu. Rev. Appl. Entomol..

[41] Asplen M.K., Anfora G., Biondi A., Choi D.-S., Chu D., Daane K.M., Gibert P., Gutierrez A.P., Hoelmer K.A., Hutchison W.D. (2015). Invasion biology of spotted wing Drosophila (*Drosophila suzukii*): A global perspective and future priorities. J. Pest Sci..

[42] Yee W.L., Alston D.G. (2006). Effects of spinosad, spinosad bait, and chloronicotinyl insecticides on mortality and control of adult and larval western cherry fruit fly (Diptera: Tephritidae). J. Econ. Entomol..

[43] Abbott W.S. (1925). A method of computing the effectiveness of an insecticide. J. Econ. Entomol..

[44] Waterfield N.R., Ciche T., Clarke D. (2009). *Photorhabdus* and a host of hosts. Annu. Rev. Microbiol..

[45] Sharad-Mohan A., Gaur H.S. (2003). Successful management of mango mealy bug, *Drosicha mangiferae* by *Photorhabdus luminescens*, a symbiotic bacterium from entomopathogenic nematode *Heterorhabditis indica*. Int. J. Nematol..

[46] Uma G.P., Prabhuraj A. (2010). Bio-efficacy of *Photorhabdus luminescens*, a symbiotic bacterium against *Thrips palmi* Karny (Thripidae: Thysanoptera). J. Biopestic..

[47] Musser F.R., Nyrop J.P., Shelton A.M. (2006). Integrating biological and chemical controls in decision making: European corn borer (Lepidoptera: Crambidae) control in sweet corn as an example. J. Econ. Entomol..

[48] Satinder K.B., Verma M., Tyagi R.D., Valéro J.R. (2006). Recent advances in downstream processing and formulations of *Bacillus thuringiensis* based biopesticides. Process Biochem..

[49] Ramesh A.K., Prabhuraj A., Ashoka J., Hanchinal S.G., Sharanagouda H. (2017). Generation and evaluation of nanoparticles of supernatant of *Photorhabdus luminescens* (Thomas and Poinar) against mite and aphid pests of cotton for enhanced efficacy. Curr. Sci..

